# TNF-α and NF-κB signaling play a critical role in cigarette smoke-induced epithelial-mesenchymal transition of retinal pigment epithelial cells in proliferative vitreoretinopathy

**DOI:** 10.1371/journal.pone.0271950

**Published:** 2022-09-01

**Authors:** Victor Wang, Alison Heffer, Elisa Roztocil, Steven E. Feldon, Richard T. Libby, Collynn F. Woeller, Ajay E. Kuriyan

**Affiliations:** 1 Flaum Eye Institute, University of Rochester Medical Center, Rochester, NY, United States of America; 2 Center for Visual Sciences, University of Rochester, Rochester, NY, United States of America; 3 Department of Environmental Medicine, School of Medicine and Dentistry, University of Rochester, Rochester, New York, United States of America; 4 Retina Service/Mid Atlantic Retina, Wills Eye Hospital, Sidney Kimmel Medical College, Thomas Jefferson University, Philadelphia, PA, United States of America; Charite Universitatsmedizin Berlin, GERMANY

## Abstract

Proliferative vitreoretinopathy (PVR) is characterized by the growth and contraction of cellular membranes within the vitreous cavity and on both surfaces of the retina, resulting in recurrent retinal detachments and poor visual outcomes. Proinflammatory cytokines like tumor necrosis factor alpha (TNFα) have been associated with PVR and the epithelial-mesenchymal transition (EMT) of retinal pigment epithelial (RPE) cells. Cigarette smoke is the only known modifiable risk factor for PVR, but the mechanisms are unclear. The purpose of this study was to examine the impact of cigarette smoke on the proinflammatory TNFα/NF-κB/Snail pathway in RPE cells to better understand the mechanisms through which cigarette smoke increases the risk of PVR. Human ARPE-19 cells were exposed to cigarette smoke extract (CSE), for 4 to 24-hours and TNFα, Snail, IL-6, IL-8, and α-SMA levels were analyzed by qPCR and/or Western blot. The severity of PVR formation was assessed in a murine model of PVR after intravitreal injection of ARPE-19 cells pre-treated with CSE or not. Fundus imaging, OCT imaging, and histologic analysis 4 weeks after injection were used to examine PVR severity. ARPE-19 cells exposed to CSE expressed higher levels of *TNFα*, *SNAIL*, *IL6* and *IL8* mRNA as well as SNAIL, Vimentin and α-SMA protein. Inhibition of TNFα and NF-κB pathways blocked the effect of CSE. *In vivo*, intravitreal injection of ARPE-19 cells treated with CSE resulted in more severe PVR compared to mice injected with untreated RPE cells. These studies suggest that the TNFα pathway is involved in the mechanism whereby cigarette smoke increases PVR. Further investigation into the role of TNFα/NF-κB/Snail in driving PVR and pharmacological targeting of these pathways in disease are warranted.

## Introduction

Proliferative vitreoretinopathy (PVR) is a condition that arises after up to 10% of rhegmatogenous retinal detachments (RRDs) and is the leading cause of RRD surgery failure [1–3]. The development of PVR is characterized by the growth and contraction of cellular membranes within the vitreous cavity and both surfaces of the retina [4]. Although many pharmacologic agents have been evaluated for inhibiting PVR in humans, no agents have been found to be consistently effective [5]. Currently, recurrent retinal detachments with proliferative vitreoretinopathy can only be managed with surgical interventions [6, 7]. Despite anatomic success after surgery, over 70% of patients have a final visual acuity of <20/400 even after successful surgical management [8]. Therefore, there is significant importance surrounding discovery of the underlying aspects of PVR mechanism to identify pharmacologic targets for prevention or treatment of PVR.

It is widely thought that after a RRD occurs, there is a breakdown of the blood-retinal barrier and migration of retinal pigmented epithelial (RPE) cells into the vitreous cavity and onto the retinal surface [1, 3, 9]. The disruption of the blood-retinal barrier enables an influx of cytokines, growth factors and infiltrating inflammatory cells into the vitreous [10–12]. These inflammatory mediators and cells interact with retinal cells and RPE cells to stimulate local production of cytokines and growth factors. This process drives the primary pathologic process of PVR, epithelial to mesenchymal transition (EMT) of RPE cells. During EMT, RPE cells start to take on a mesenchymal morphology and express filamentous proteins such as alpha smooth muscle actin (αSMA) [13, 14]. This transformation into contractile fibrotic cells results in exertion of tractional forces on the retina, resulting in the recurrent RDs secondary to PVR [11].

Cigarette smoke has been associated with higher rates of PVR and is currently the only known modifiable risk factor for PVR. The mechanisms whereby cigarette smoke increase PVR are unclear [15, 16]. In lung epithelial cells, cigarette smoke increases EMT through activating the tumor necrosis factor alpha (TNFα)/NF-κB/Snail pathway and increasing production of the proinflammatory cytokines IL-6 and IL-8 [17–20]. Snail is a key transcription factor that induces EMT and previous studies have also revealed that IL-6 and IL-8 promote EMT [21, 22]. TNFα activates the canonical NF-κB pathway, which in turn increases Snail expression through multiple mechanisms [23, 24].

TNFα has been identified as a key cytokine involved in driving PVR. TNFα is elevated in human vitreous and epiretinal membranes of patients with PVR [25–28]. Additionally, polymorphisms within the TNFα locus were significantly associated with PVR [29]. *In vitro*, TNFα induces RPE cells to upregulate EMT markers and activate the mesenchymal phenotype by aggregating through contraction-dependent mechanisms [30, 31]. Additionally, TNFα levels are increased in the vitreous of both mouse and rabbit PVR models [32, 33]. Inhibition of TNFα via substance P prevents RPE cell EMT *in vitro* and PVR formation *in vivo* [34].

Based on these studies, we investigated whether cigarette smoke stimulates the TNFα/NF-κB/Snail pathway in RPE cells to assess whether these pathways could be involved in the pathogenesis of the increased risk of PVR associated with cigarette smoke. Here, we report that cigarette smoke extract (CSE) upregulates both proinflammatory cytokines and EMT markers in ARPE-19 cells. Inhibition of the TNFα and NF-κB pathways blocks CSE-induced proinflammatory cytokine production and EMT in ARPE-19 cells. Furthermore, we show that injecting CSE-treated ARPE-19 cells significantly increases PVR severity and EMT marker production over untreated cells in a mouse model of PVR.

## Materials and methods

### Cell culture

Human ARPE-19 cells (ATCC, Manassas, VA) were cultured in Dulbecco’s Modified Eagle’s Medium (DMEM) and Ham’s F12 media (1:1) supplemented with 10% fetal bovine serum (FBS; Hyclone, Chicago, IL) and 1% anti-anti (ThermoFisher, Waltham, MA) at 37°C and 5% CO_2_. ARPE-19 cells between passages 3 and 12 were plated in 6-well plates at a density of 20,000 cells/cm^2^ for 24 hours. Cultures were placed in DMEM/F12 + 0.1% FBS for 18 hours before treatment to reduce serum factor responses. To investigate the role of TNFα, serum starved ARPE-19 cells were pre-treated with 1μM Cas 1049741 (Calbiochem, San Diego, CA) in DMEM/F12 + 0.1% FBS for one hour at 37°C. The media was then replaced with 0% or 1% CSE combined with 1uM Cas 1049741 for 4 or 24 hours at 37°C. To investigate the role of NF-κB pathway, serum starved ARPE-19 cells were incubated with 1μM Bay-11-7082 (Enzo Life Sciences, Farmingdale, NY) in DMEM/F12 + 0.1% FBS for one hour before treatment with 0% or 1% CSE combined with 1uM Bay-11-7082 for 4 or 24 hours at 37°C.

### Cigarette smoke extract

Cigarette smoke extract (CSE) was prepared similarly to Bertram et. al, 2009 [35]. One 1R3F research-grade cigarette (University of Kentucky) was bubbled through 10mL DMEM/F12 + 0.1% FBS using a vacuum apparatus at a burn rate of 2 minutes per cigarette. The resulting CSE was pH-adjusted to 7.3 and filtered through a 0.2 μm pore filter (GE Healthcare Life Science, Marlborough, MA). CSE was standardized by measuring its UV light absorbance at 320 nm on a Denovix DS-11 Spectrophotometer and diluted with DMEM/F12 + 0.1% FBS until an absorbance of 0.65 ± 0.03 was achieved; this stock was defined as 10% CSE and diluted further in starved media for all experiments.

### Western blotting

After 4 or 24 hours of treatment, cells were washed with PBS, and lysed with 60mM Tris-HCl (pH 6.8) with 2% SDS and 1x protease inhibitor mixture (Sigma, St. Louis, MO). Lysates were sonicated for 5 seconds to shear genomic DNA and the resulting total protein concentration was measured using a detergent-compatible (DC) protein assay (Bio-Rad, Hercules, CA). 10 μg total protein for each sample was separated with a 4–20% TGX gradient gel (Bio-Rad, Hercules, CA) and transferred to 0.45 μm PVDF membrane (Millipore, Burlington, MA). Primary antibodies used for detection were αSMA (mouse, 1:6000, Sigma, St. Louis, MO), αSnail (rabbit, 1:1000, Cell Signaling Technologies, Danvers, MA), and β-tubulin (rabbit, 1:1000, Cell Signaling Technologies, Danvers, MA). HRP-conjugated secondary antibodies (1:5000, Jackson ImmunoResearch, West Grove, PA) were used and detected using Western-Lightning Plus-ECL (Perkin Elmer, Waltham, MA). ImageLab (Bio-Rad, Hercules, CA) was used to quantify band intensities with expression levels for each primary antibody normalized to β-tubulin.

### RNA isolation and RT-qPCR analysis

At the desired timepoint, cells were washed with PBS, and total RNA was extracted from cells using TRIzol reagent per the manufacturer’s protocol (Invitrogen, Waltham, MA). RNA concentration and quality measured on a Denovix DS-11 Spectrophotometer. cDNA was synthesized from 1 ug total RNA using QuantiTect Reverse Transcription Kit (Qiagen, Hilden, Germany) and 25 ng cDNA was used in each reaction. Each set of reactions was run in triplicate using SsoAdvanced Universal SYBR Green Supermix (Bio-Rad, Hercules, CA). Calculations for relative expression levels were performed using comparative CT method [36], with gene expression levels normalized to GAPDH.

Primers used included:

**Table:** 

Primer	Forward	Reverse
*GAPDH*	5’-GACCCTCACTGCTGGGGAGT-3’	5’-GATGGTACATGACAAGGTGCGGC-3’
*TNFα*	5’-ACTTTGGAGTGATCGGCC-3’	5’-GCTTGAGGGTTTGCTACAAC-3’
*Snail*	5’-CCCTCAAGATGCACATCCGAA-3’	5’-GACTCTTGGTGCTTGTGGAGCA-3’
*IL6*	5’-GTACATCCTCGACGGCATC-3’	5’-ACCTCAAACTCCAAAAGACCAG-3’
*IL8*	5’-GTGTAAACATGACTTCCAAGCTG-3’	5’-GTCCACTCTCAATCACTCTCAG-3’

### Use of animals

A total of 44 eight to ten-week-old female C57BL/6J mice were purchased from Jackson Laboratory (Bar Harbor, ME). All experiments adhered to ARVO Statement for the Use of Animals in Ophthalmic and Vision Research and were approved by the University Committee of Animal Resources (UCAR) of the University of Rochester. Mice were anesthetized with 100mg/kg ketamine (Par Pharmaceuticals, Chestnut Ridge, NY) and 10mg/kg xylazine (Akorn Inc, Lake Forest, IL). The eye being injected was sterilized with 5% betadine diluted in saline. An initial puncture through the sclera into the vitreous cavity was performed with a 30G needle just posterior to the corneal-scleral junction. 0.5μL SF_6_ gas (Alcon Laboratories, Ft. Worth, TX) was injected using a 33G needle on a Hamilton syringe (Hamilton, Reno NV) into the vitreous cavity through the scleral puncture wound made by the 30G needle, taking special care not to damage the lens as previously described [37]. One week later, RPE cells cultured for 24 hours in either DMEM/F12 + 0.1% FBS (control) or 0.5% CSE (treated) were counted using TC-20 Automatic Cell Counter (Bio-Rad, Hercules, CA) and resuspended in DMEM/F12 + 0.1% FBS or 0.5% CSE, respectively, to a final concentration of 2000 cells/μL. There were three conditions used for intravitreal injection: 1) RPE cells treated for 24 hours and resuspended in DMEM/F12 + 0.1% FBS (n = 19), 2) RPE cells treated and resuspended in 0.5% CSE (n = 16), 3) 10% CSE alone (n = 9). 1μL of RPE cells or 10% CSE was injected within an hour of cell collection, with mice anesthetized and prepared similarly as for the SF_6_ injection. Immediately prior to injection, RPE cells were gently agitated to resuspend them, and 1μL was loaded into a 10μL Hamilton syringe using a 33G needle as previously described [37]. The same scleral site punctured with the 30G needle for the SF6 gas injection the prior week was re-punctured with a 30G needle to minimize scarring. The RPE cells were slowly injected with the 33G needle and the needle was left in the eye for 30 seconds to minimize cells refluxing after needle removal. Animals were euthanized using compressed CO_2_ gas followed by cervical dislocation, according to the American Veterinary Medical Association Guidelines for Euthanasia of Animals: 2020 Edition. After euthanasia, whole eyes were harvested by placing curved forceps into the orbit behind the globe of the eye to collect the entire eyeball. Once isolated, any connective tissue was removed and then the eye was processed as described below.

### Fundus imaging

After RPE injection, PVR development and progression was monitored with weekly fundus imaging. Prior to imaging, mice were anesthetized with 100mg/kg ketamine and 10mg/kg xylazine. Pupils were dilated with topical 2.5% phenylephrine (Paragon Bioteck Inc, Portland, OR) and 1% tropicamide (Akorn Inc, Lake Forest, IL). After waiting for pupillary dilation, a mouse was positioned for imaging and GenTeal lubrication gel (Alcon, Fort Worth, TX) was applied to cornea to prevent drying of ocular surface. Imaging was performed using bright-field view of Micron III (Phoenix Instruments, Naperville, IL). The camera was placed just above surface of cornea and fundus images were taken using StreamPix software (Norpix, Montreal, Quebec). If imaging showed media opacities that prevented a clear view of the retina, the image was considered ungradable and excluded from analysis.

### Optical Coherence Tomography (OCT) imaging

Mice were imaged with OCT to monitor changes in retinal structure as well as the presence of vitreous and pre-retinal cells/membranes. Prior to imaging, anesthetization, pupil dilation and GenTeal were applied as with fundus imaging. Each mouse was positioned in a holder with bite-bar stabilization of head and had a small contact lens placed on the eye to improve optics of OCT. Heidelberg Spectralis HRA+OCT imaging system (Heidelberg Engineering, Franklin, MA) was used to capture OCT images.

### Histologic analysis

Whole eyes were harvested in the third (n = 5 per condition) or fourth week post-RPE injection and fixed in 4% paraformaldehyde for 24 hours at 4°C. The eyes were then treated with a series of ethanol and xylene washes before being embedded in paraffin. A Micron HM310 device was used to obtain paraffin sections of 10μm thickness and dried on SuperFrost Plus slides (Fisher, Waltham, Massachusetts). Hematoxylin and eosin (H&E) staining was done after deparaffinization and rehydration of slides through a sequence of xylene and ethanol washes. Slides were stained with Mayer’s Hematoxylin Solution (Sigma, St. Louis, MO) for 2–3 minutes, incubated in Bluing Reagent (0.1% NaHCO3) for 30 seconds and rinsed twice in ethanol before counterstained with Eosin Y (Sigma) for 2 minutes. Slides were mounted with Permount (Electron Microscopy Biosciences, Hatfield, PA) and imaged using Olympus bx51 microscope (Olympus Life Science, Waltham, MA) at 10x magnification. For immunohistochemical staining, Slides were deparaffinized and rehydrated using xylene, ethanol, and then water washes. For antigen retrieval, slides were boiled gently in Citrate-based Antigen Unmasking Solution (Vector Laboratories, Burlington, Ontario) for 5 minutes in the microwave. The slides were then washed in Tris-buffered saline/Triton-X, blocked with 10% goat serum/1% bovine serum albumin for 2 hours at room temperature, and then incubated overnight in primary antibody diluted with 5% bovine serum albumin in Tris-buffered saline. Slides were rinsed in Tris-buffered saline/Triton-X and incubated in 0.3% H_2_O_2_ for 15 minutes to block endogenous peroxidase activity before incubation in a horseradish peroxidase-conjugated secondary antibody diluted in 5% bovine serum albumin for 2 hours at room temperature. The slides were washed and stained with diaminobenzidine (Vector laboratories) until color was visible. The slides were then counterstained with hematoxylin, dehydrated, cleared, and mounted with Permount (Electron Microscopy Biosciences, Hatfield, PA) and imaged using Olympus bx51 microscope. Primary antibodies and dilutions used were αSMA (1:250, rabbit, Abcam, Cambridge, UK) and αVimentin (1:250, rabbit, Cell Signaling Technologies, Danvers, MA). The secondary antibody used was a horseradish peroxidase-conjugated antibody (1:500, goat, Jackson ImmunoResearch, West Grove, PA).

### Statistical analysis

Data is presented as the mean ± standard error. Findings from *in vitro* experiments were replicated at least 3 times using ARPE-19 cells at different passages. Statistical significance was determined using two-sample T-test assuming unequal variances with p values <0.05 considered significant. Fundus images of the mice collected post-RPE injection were graded according to a previously established grading scale appropriate for the mouse [37]. The distribution of grades for each injection group are presented as violin plots (GraphPad Prism). Mann-Whitney U Test was completed as statistical analysis for comparing mean PVR grade between groups sacrificed at four weeks. A p-value <0.05 was considered significant.

## Results

### CSE exposure induces TNF-α and Snail mRNA and protein expression

**Fig 1:**
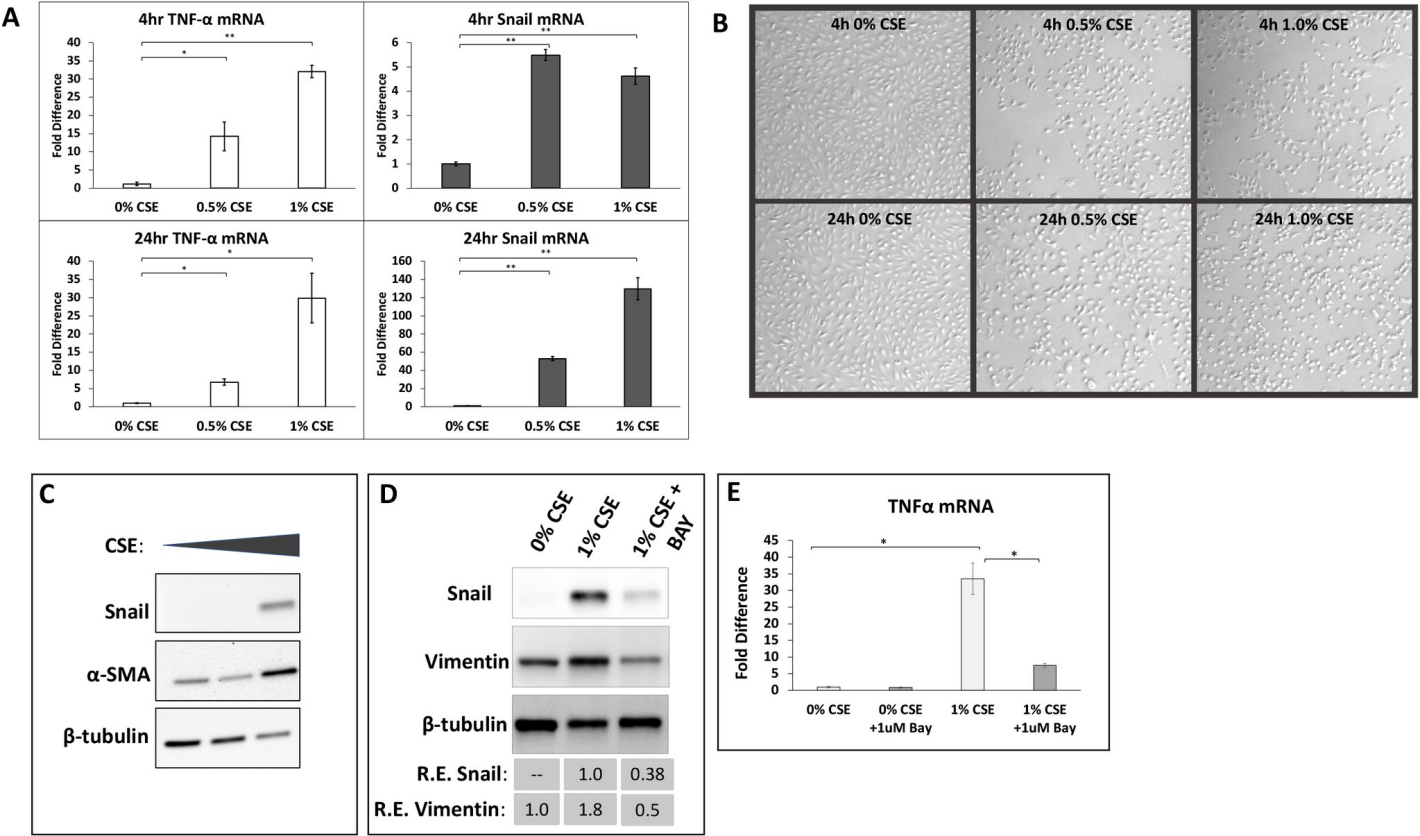
Cigarette smoke extract (CSE) increases TNFα and EMT markers in ARPE-19 cells. (A) ARPE-19 cells were treated with untreated culture medium (0% CSE) or 0.5% or 1% CSE in culture medium for 4 or 24 hours. After treatment, cells were collected and RNA was isolated. Exposure to 0.5 or 1% CSE for 4 or 24 hours induced expression of TNFα (left) and Snail (right) mRNA. mRNA levels are normalized to GAPDH mRNA levels and presented as fold change over untreated cells(0% CSE). Error bars represent standard error of the mean, with N = 3 for each point. * = p<0.05 and ** = p<0.01 compared to 0% CSE. (B) Representative cell culture images of ARPE-19 cells without CSE (0% CSE) or with 0.5 or 1% CSE at 4 and 24 hours. CSE alters the morphology of the ARPE-19 cells to become more rounded, especially at 24 hours treatment. (C) ARPE cells were treated with or without CSE as in (A) for 24 hours. Afterwards, cells were lysed and protein expression was analyzed by Western blot. CSE elevates both αSMA and SNAIL levels. (D) ARPE-19 cells were treated with 1% CSE in the presence or absence of 1 uM of the NF-κB inhibitor, BAY-11-7082 (BAY). After 24 hours, cells were harvested and protein and mRNA levels were analyzed as described above. Inhibition of NF-κB mitigated the ability of CSE to induce SNAIL and EMT marker, vimentin. (E) RNA was isolated from cells treated as in (D). BAY-11-7082 mitigated the ability of CSE to induce *TNFα* mRNA.

Cigarette smoke extract (CSE) stimulated ARPE-19 cells induce *TNFα* mRNA expression and a key transcription factor associated with EMT, *SNAIL*. A dose-dependent effect was observed for *TNFα* and *SNAIL* at both 4 and 24-hour timepoints as shown by qPCR analysis (Fig 1A). Treatment with a lower concentration of CSE (0.5% CSE) stimulated a 14-fold increase in *TNFα* expression at 4 hours (p<0.05) and a 7-fold increase at 24 hours (p<0.05), while treating with a higher concentration (1% CSE) led to a 32-fold increase at 4 hours (p<0.01) and 30-fold increase by 24 hours (p<0.05). Therefore, *TNFα* mRNA levels appear to decrease slightly between 4 and 24-hours. Treatment of ARPE-19 cells with 0.5% CSE produced a 5.5-fold increase in *SNAIL* mRNA levels at 4 hours, which increased to a 53-fold increase by 24 hours. Stimulation with 1% CSE also induced significant elevations in *SNAIL* mRNA: 4.6-fold after 4 hours and 130-fold increase after 24 hours. After CSE exposure, changes in the morphology of ARPE-19 cells were observed (Fig 1B). At both 4- and 24-hour timepoints, 0.5% and 1% CSE exposure caused the cells to take on a more rounded appearance compared to untreated (0% CSE) ARPE-19 cells. The most pronounced changes in morphology were observed at 24 hours with 1% CSE (bottom right image, Fig 1B). Western blot analysis was consistent with qPCR results, showing elevated SNAIL protein levels 24 hours after CSE treatment (Fig 1C). Western blotting revealed that αSMA, a key marker of EMT, was increased by 1.0% CSE treatment. 1.0% CSE also induced vimentin expression in ARPE-19 cells, further suggesting CSE induces EMT (Fig 1D). The results of these experiments show CSE-induces *TNFα* and *SNAIL*, which likely play an important role in driving EMT. In order to investigate the mechanism whereby CSE induced *TNFα* expression, the NF-kB pathway inhibitor, BAY-11-7082, which targets IκBα kinase to prevent NF-kB signaling [38], was used (Fig 1D and 1E). BAY-11-7082 treatment attenuated the ability of CSE to induce SNAIL and vimentin protein (Fig 1D). Furthermore, treatment of BAY-11-7082 led to a significant reduction in *TNFα* mRNA levels compared to CSE alone suggesting that *TNFα* mRNA expression and EMT is induced by NF-kB pathway.

### Inhibition of TNFα or NF-κB downregulates SNAIL and inflammatory cytokine levels in ARPE-19 cells

**Fig 2:**
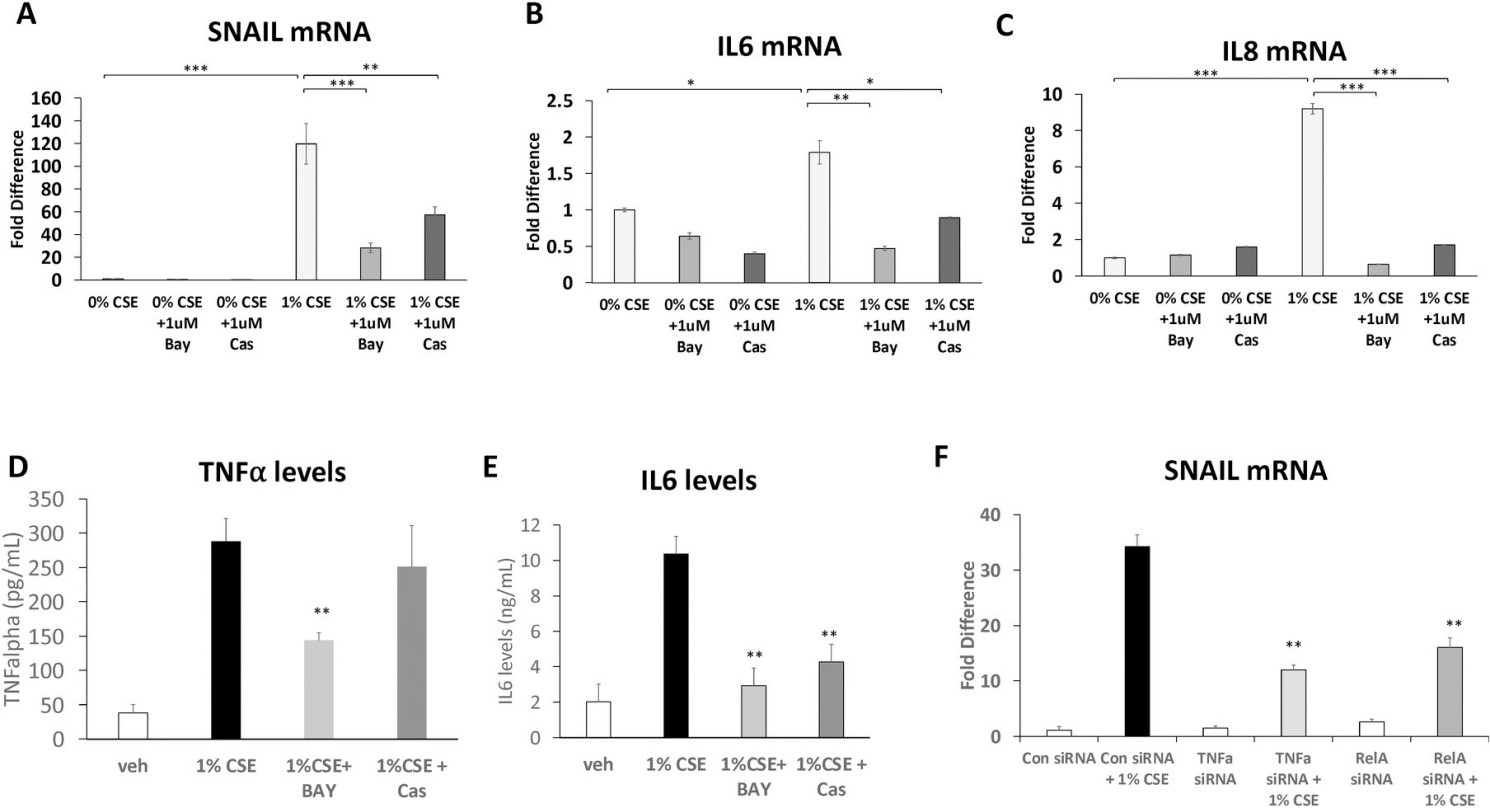
Inhibition of the TNFα-dependent NF-κB pathway blocks SNAIL and inflammatory cytokine production in CSE exposed ARPE-19 cells. ARPE-19 cells exposed to CSE for 24 hours in the presence or absence of the NF-κB inhibitor, BAY-11-7082 (BAY, 1 μM) or the TNFα inhibitor, CAS 1049741 (CAS, 1 μM). After exposure, total RNA was isolated and analyzed by qPCR. CSE exposed ARPE-19 cells produced elevated *SNAIL* (A), *IL6* (B), and *IL8* (C) mRNA. Fold differences were normalized to 0% CSE for each condition. (D) TNFα and (E) IL-6 production from culture supernatants from cells treated as above were analyzed by ELISA. CSE exposure increased both TNFα and IL-6. Inhibition of NF-κB with BAY decreased TNFα production in ARPE-19 cells cultured with CSE while CAS did not. Both BAY and CAS significantly decreased IL-6 production. (F) APRE-19 cells were treated with control, TNFα or RELA siRNA for 24 hours. After treatment with siRNA, cells were exposed, or not, to 1.0% CSE for 24 hours. After exposure, cells were harvested and RNA levels were analyzed by qPCR. *SNAIL* mRNA levels were significantly increase by CSE exposure in control siRNA treated cells while SNAIL mRNA production was significantly attenuated in cells cultured with TNFα or RELA siRNA. Error bars represent standard error of data set. N≥3 for each. *p<0.05, **p<0.01, ***p<0.001.

CSE stimulation of ARPE-19 cells for 24 hours induced *SNAIL* mRNA by 120-fold relative to control (p<0.001, Fig 2A). Bay-11-7082 decreased the CSE-induced expression of *SNAIL* by 4-fold (p<0.001). To further define the role of TNFα signaling in driving SNAIL expression in ARPE-19 cells, the small molecule TNFα inhibitor, CAS 1049741 (CAS) was used [39]. CAS disrupts the ability of TNFα to form a functional trimer molecule and thus prevents TNFα binding to its receptor. CAS selectively inhibits TNFα and not IL-1beta-induced NF-κB activity [39]. Our results show that SNAIL levels are reduced by CAS 1049741 (Fig 2A) suggesting that SNAIL is induced specifically by TNFα induced NF-κB activity. Following a similar pattern, ARPE-19 cells exposed to CSE had a 1.8-fold higher *IL6* expression relative to control media (p<0.05, Fig 2B). Both BAY and CAS attenuated CSE induced *IL6* mRNA. ARPE-19 cells cultured in CSE had a 9.2-fold increase in *IL8* mRNA expression relative to control (p<0.001, Fig 2C). As with *SNAIL* and *IL6*, the upregulation of *IL8* was blocked by Bay-11-7082 (15-fold decrease, p<0.001) or Cas (5-fold decrease, p<0.001). As further evidence that CSE induces TNFα and IL-6 production, we measured their production by ELISA (Fig 2D and 2E). TNFα and IL-6 levels were significantly increased by CSE treatment. BAY-11-7082 reduced TNFα and IL-6 levels while CAS blocked IL-6 production but not TNFα. To complement the pharmacological approach of TNFα and NF-κB inhibitors, we used siRNA to knockdown either TNFα or RELA (also called p65, an essential NF-κB subunit) in the presence or absence of 1.0% CSE in ARPE-19 cells (Fig 2F). Knockdown of TNFα or RELA using siRNA significantly attenuated the induction of SNAIL mRNA by CSE. These results suggest that CSE induces SNAIL and EMT transition through a TNFα-dependent NF-κB pathway.

### Intravitreal injection of RPE cells exposed to cigarette smoke induced more severe PVR

**Fig 3:**
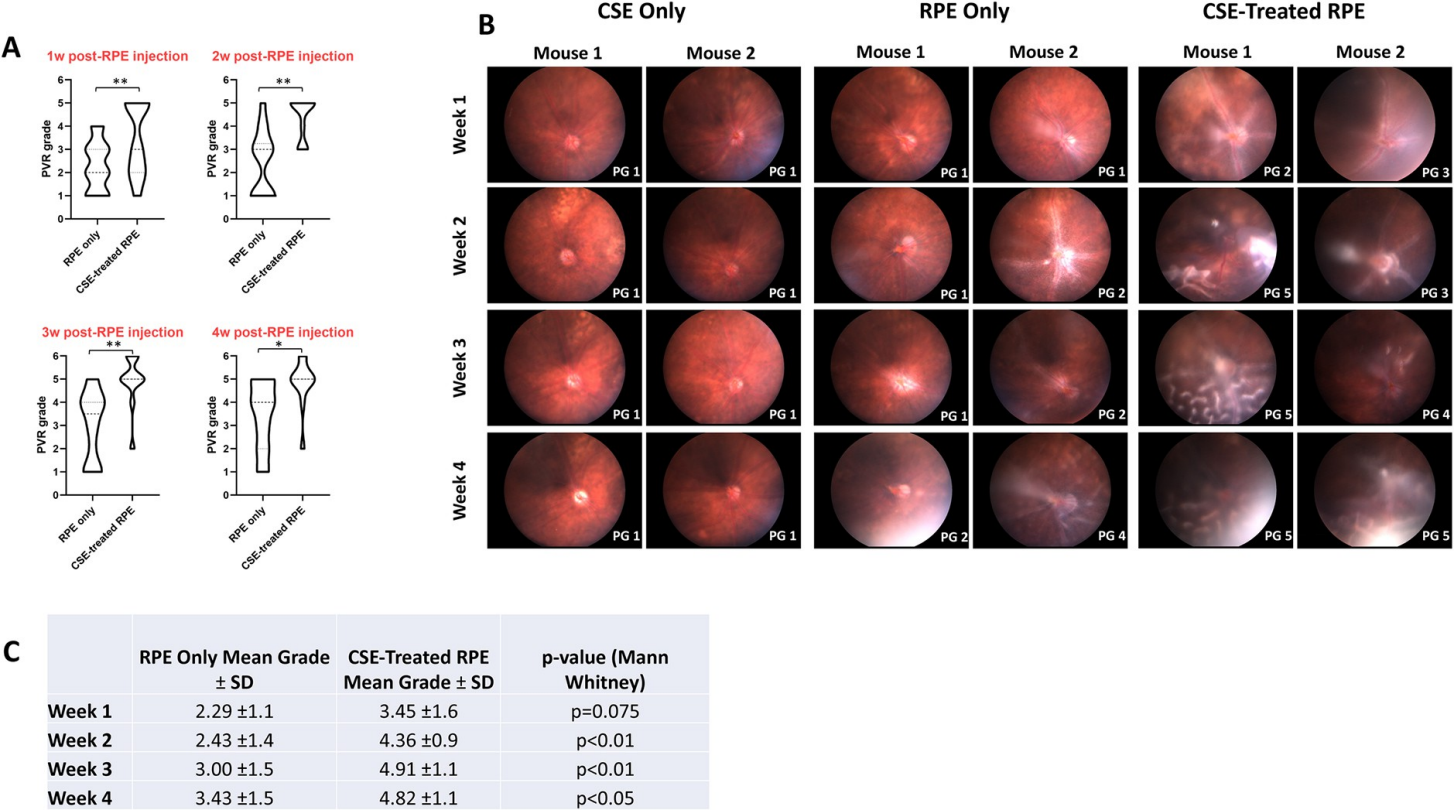
Intravitreal injection in mice with CSE-treated RPE cells resulted in more rapid and severe PVR development. RPE only injections consisted of 2,000 ARPE-19 cells resuspended in 1uL media (N = 14). CSE-treated RPE injections consisted of 2,000 ARPE-19 cells cultured and resuspended in 0.5% CSE (N = 11). PVR grades are based on fundus photography. (A) Both RPE only and CSE-treated RPE injected eyes developed more severe PVR over time, although CSE-treated RPE injected mice developed significantly worse PVR at every timepoint. (B) Representative fundus images of eyes injected with CSE only, RPE only, or CSE-treated RPE at each timepoint. Assigned PVR grade (PG) is listed bottom right of each image. (C) Table comparing mean PVR grades of eyes injected with RPE only or CSE-treated RPE cells.

To study the effect of cigarette smoke on severity of PVR development, ARPE-19 cells that had been treated with CSE for 24 hours were injected into the mouse vitreous and compared to control eyes injected with untreated ARPE-19 cells as previously described (Fig 3C) [37]. PVR development was graded based on weekly fundus imaging (Fig 3A and 3B) [37]. At one-week post-RPE injection, mean PVR grade was 2.29±1.1 in control RPE-injected mice while CSE-treated RPE-injected mice developed mean PVR grade of 3.45±1.6 (NS) (Fig 3C). By two weeks post-RPE injection, mean PVR grade was 2.43±1.4 in control RPE mice and 4.36±0.9 in CSE-treated RPE mice (p<0.01). By three weeks, mean PVR grade was 3.00±1.5 in control RPE mice and 4.91±1.1 in CSE-treated RPE mice (p<0.01). Finally, by four weeks, mean PVR grade was 3.43±1.5 in control RPE mice and 4.82±1.1 in CSE-treated RPE mice (p<0.05). In summary, ARPE-19 cells treated and cultured with CSE (n = 11) developed more rapid and severe PVR compared to ARPE-19 cells in control media (n = 14). Unlike RPE or CSE-stimulated RPE cells, intravitreal injections of CSE alone did not result in PVR development (Fig 3B), despite a high concentration of CSE used (10% CSE) demonstrating that CSE alone does not promote PVR formation (n = 9).

**Fig 4:**
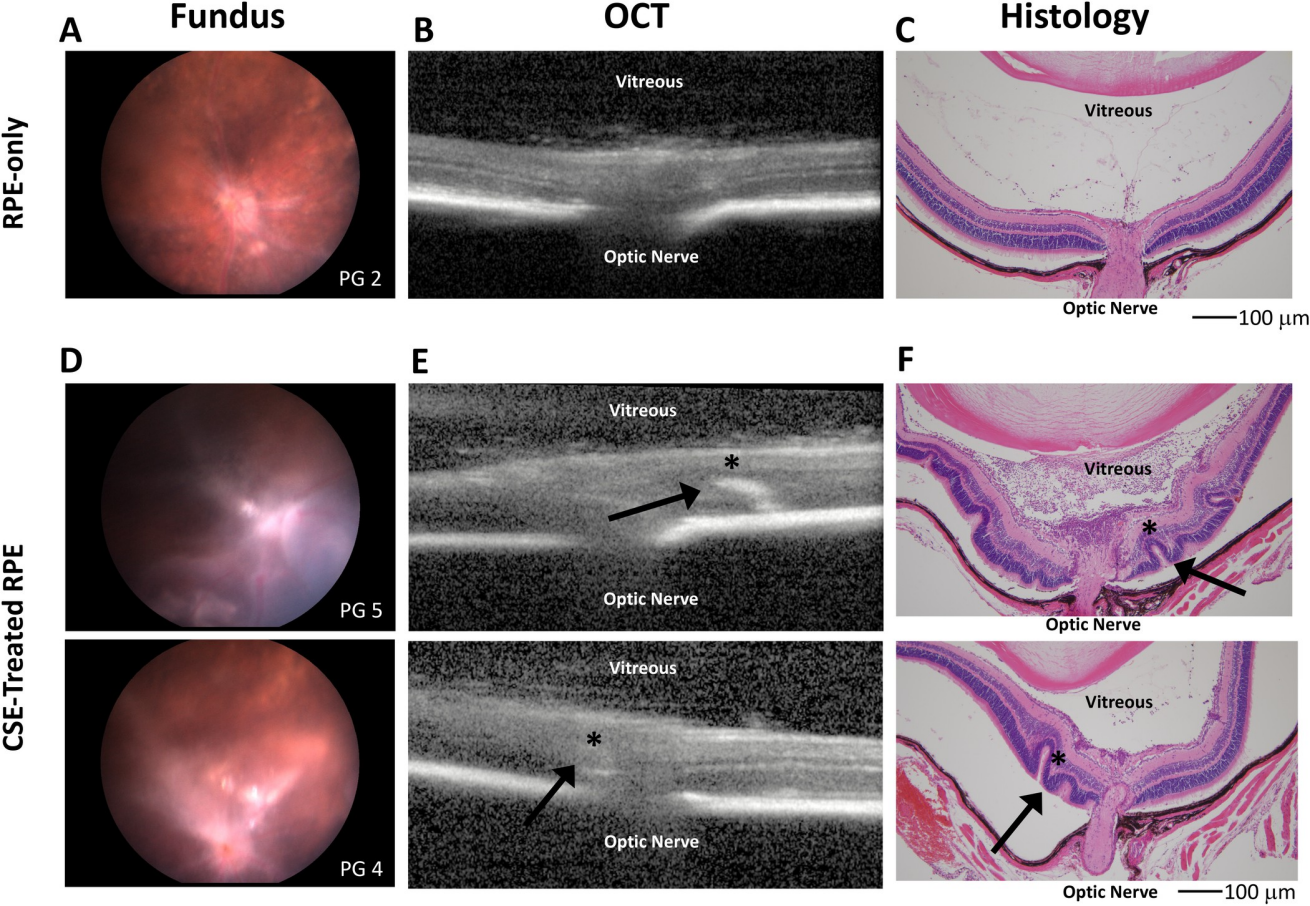
CSE treated RPE cell injection increased retinal folds, retinal detachment and PVR membranes thickness compared to control RPE. Fundus imaging, OCT, and histology of representative eyes from mice injected intravitreally with control RPE cells (A,B,C) vs. RPE cells treated and resuspended with 0.5% CSE (D,E,F). All images were taken at 4 weeks post-RPE injection. Intravitreal injection of CSE-treated RPE resulted in development of more severe PVR by 4 weeks with evidence of retinal folding, significant areas of detachment, and inflammatory infiltrate. The inflammatory infiltrate is especially prevalent in the vitreous of the top section in F. Arrows and * in E and F highlight retinal folds observed on OCT imaging seen in histologic cross-sections. Scale bars represent 100 microns (μm) in length.

We used histological analysis to confirm that CSE promoted more severe PVR formation (Fig 4). Intravitreal injection of RPE cells in control media showed a thickening of the retina near the optic nerve as well as PVR membranes throughout the vitreous (Fig 4A–4C). Mice that received intravitreal injection of RPE cells pre-treated with CSE had several retinal folds, significant detachments, and PVR membranes that developed along the inner retinal surface (Fig 4D–4F).

**Fig 5:**
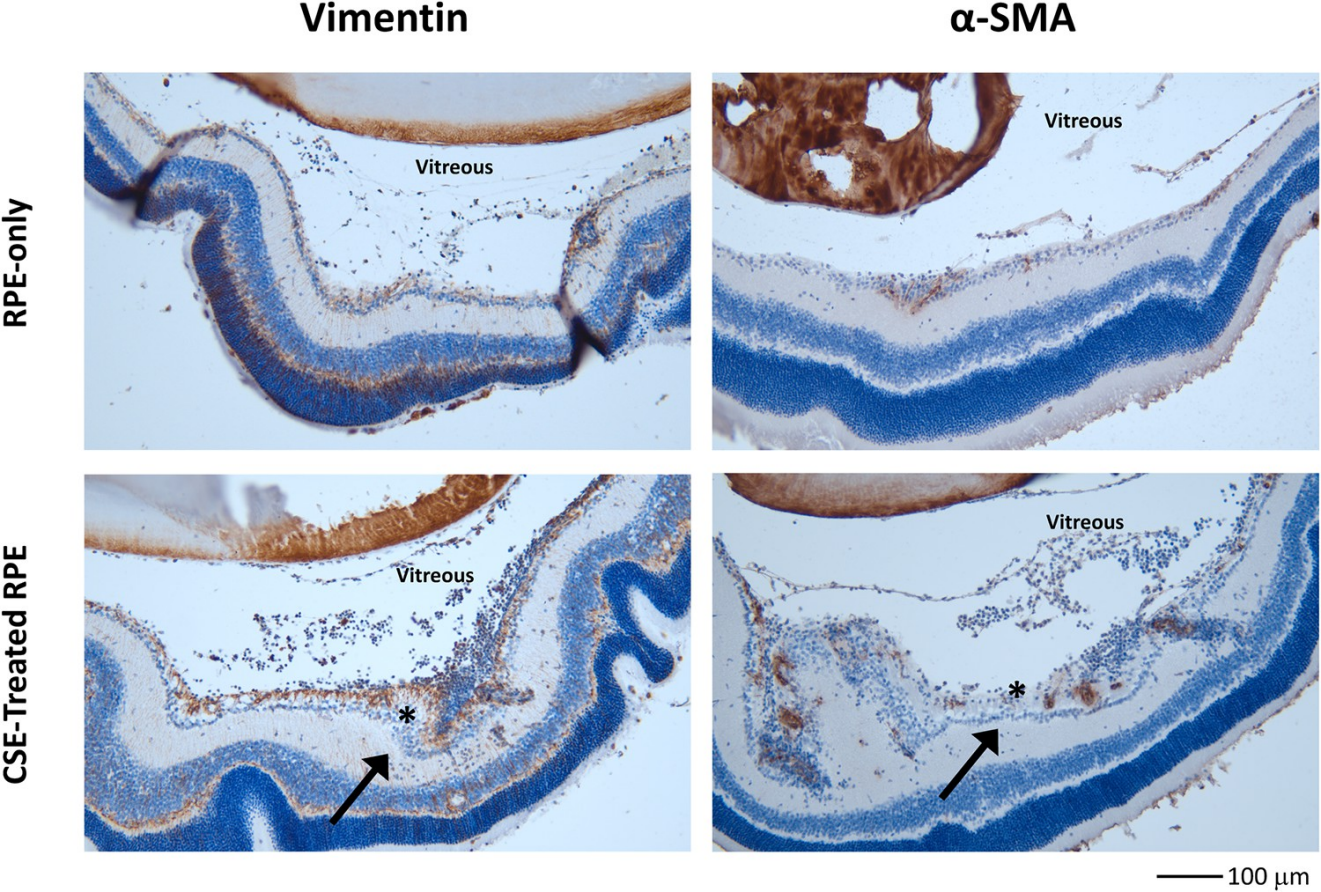
CSE treated RPE cells increased expression of Vimentin and αSMA in PVR compared to control RPE cells. Immunohistochemistry sections of representative eyes from mice injected intravitreally with control RPE cells vs. RPE cells treated and resuspended with 0.5% CSE. Like in Fig 4, images were taken at 4 weeks post-RPE injection. Intravitreal injection of CSE-treated RPE resulted in significantly elevated levels of vimentin and αSMA protein expression (brown pigment) in both vitreous and retinal tissue. The arrows and * highlight increases in the amount of cellularity and in the amount of vimentin and αSMA staining in and near the retina in CSE-treated samples. The scale bar at the bottom right represents 100 microns (μm) in length.

Immunohistochemistry analysis demonstrated robust pre-retinal and intraretinal expression of EMT markers Vimentin and αSMA in mouse eyes that underwent intravitreal injection of ARPE-19 cells cultured and suspended in CSE media (Fig 5). Arrows highlight areas of increased accumulation of vimentin and αSMA in the bottom panels of Fig 5. In comparison, mouse eyes that underwent intravitreal injection of RPE cells cultured in control media had limited pre-retinal and intra-retinal expression of both Vimentin and α-SMA. Together, the results show intravitreal injection of RPE cells cultured with and resuspended in CSE stimulates increased formation of PVR membranes, retinal folds, and upregulation of EMT markers, similar to findings of more severe PVR seen in humans [12].

## Discussion

Nearly 14% of U.S. adults currently smoke cigarettes [40]. Cigarette smoke accounts for 20% of deaths every year and has been associated with a wide array of diseases, from depression and cancer to shortening of telomeres and ocular diseases [41–45]. Smoking is the only known modifiable risk factor for PVR, although the mechanisms are unclear [15]. In other cell types and systems, cigarette smoke has been shown to elevate TNFα levels and induce EMT [17, 19, 46]. Our study found that CSE similarly induces RPE cell EMT and increases TNFα levels.

The increased production of TNFα by ARPE-19 cells when cultured with CSE suggests activation of proinflammatory pathways [47]. *TNFα* mRNA production peaked at 4 hours post CSE and demonstrates that *TNFα* is produced rapidly as seen in lung epithelial cells [48]. Given the role of Snail as a crucial transcription factor that drives EMT [17], the upregulation of Snail mRNA and protein after CSE stimulation of ARPE-19 cells suggests activation of EMT. This is supported by our findings (Fig 1C) and previous reports of CSE-induced increased expression in αSMA and other markers of EMT [14]. Together, the production of TNFα and Snail with CSE stimulation, suggest a link between TNFα and CSE-induced EMT in RPE cells. While we show increased vimentin and αSMA protein expression as surrogates of EMT in CSE exposed ARPE-19 cells, there are also additional functional effects of EMT such as cellular migration. Cell migration can often be assed using assays such as scratch-migration assays. However, while CSE can initiate EMT marker expression, CSE also induces alterations in cell morphology and can induce cell death and slows proliferation *in vitro*. For example, our morphological changes observed in Fig 1B, specifically, 0% CSE vs 1.0% CSE at 24 hr timepoint. Thus, it is challenging to demonstrate efficacy in functional scratch assays *in vitro*.

**Fig 6:**
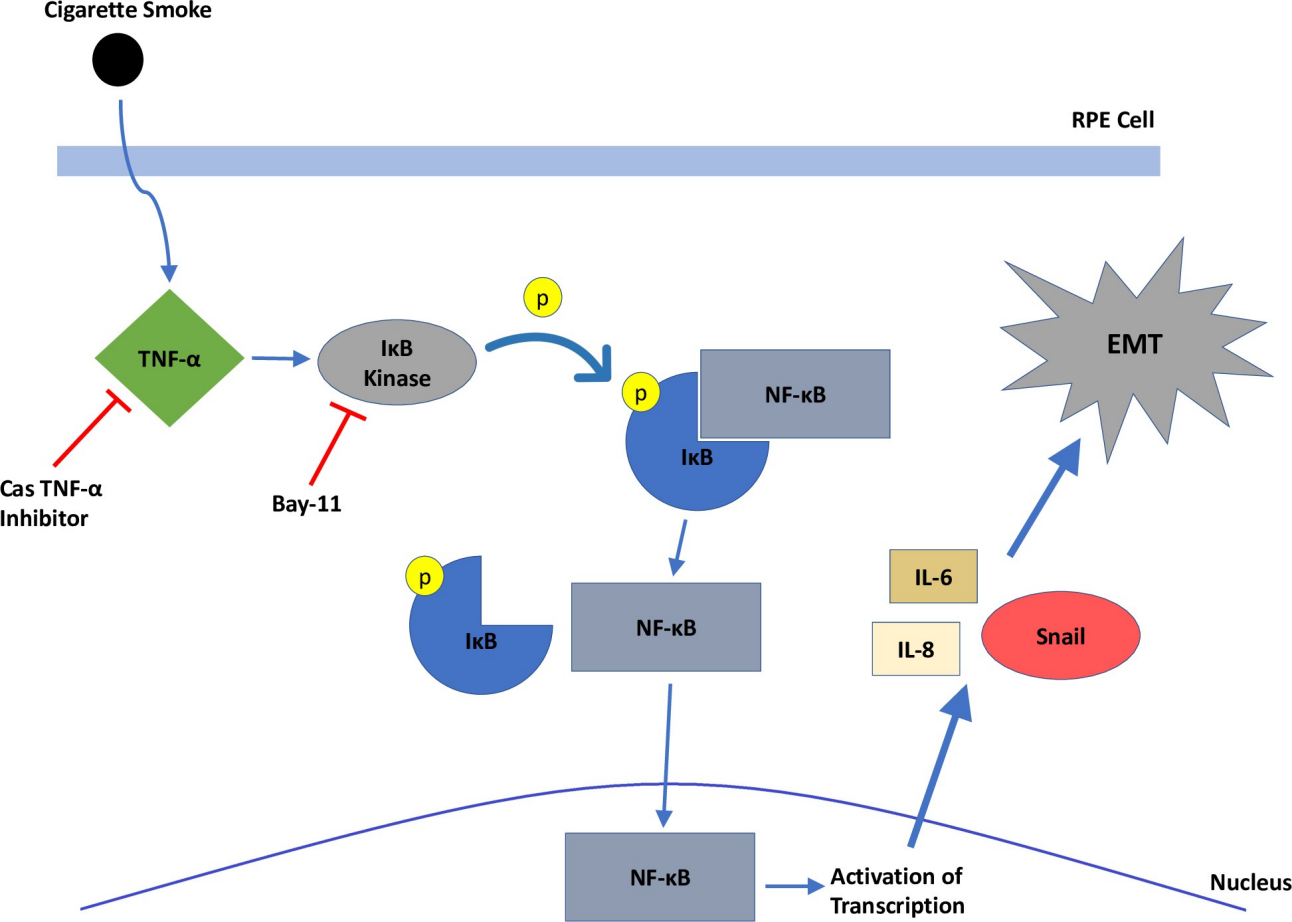
A model of cigarette smoke mechanism of action on RPE cells. Cigarette smoke stimulates production of TNF-α intracellularly, which activates IκB kinase, phosphorylating IκB. This results in dissociation of IκB from NF-κB, which enters the nucleus to activate transcription of Snail, IL-6, and IL-8. Snail triggers downstream factors involved in epithelial-mesenchymal transition.

The inhibition of CSE-induced ARPE-19 cell *IL-6* and *IL-8* production by an TNFα inhibitor, CAS, and an NF-κB inhibitor targeting IκBα kinase, BAY, suggests the TNFα/NF-κB pathway plays an important role in this process [49]. The inhibition of SNAIL protein and mRNA levels by blocking TNFα/NF-κB pathways in CSE-stimulated cells is consistent with our hypothesis that EMT is driven through the TNFα signaling (Fig 6). This association is also supported by prior studies showing that IL-6 and IL-8 upregulate EMT [22, 23]. These findings indicate that a likely mechanism of cigarette smoke stimulating EMT in ARPE-19 cells through TNFα, which activates the NF-κB pathway, leading to downstream activation of Snail and EMT. This is consistent with studies in other organ systems, such as in the lung where cigarette smoke promoted EMT through the TNFα/NF-κB/SNAIL pathway [17]. The finding that intravitreal injection of CSE-treated ARPE-19 cells upregulated EMT markers and induced more rapid and severe PVR development than RPE cells in control media is consistent with the *in vitro* finding that CSE is contributing to the promotion of EMT.

The finding that cigarette smoke may induce PVR through a TNFα/NF-κB-driven mechanism has significant therapeutic implications. Development of a new drug or treatment is an expensive and long process. However, there are established agents that inhibit TNFα and NF-κB for other chronic conditions [23, 50]. Recombinant antibodies targeting TNF-α have been commercially available for years, to treat a wide array of autoimmune diseases [51–53]. MicroRNAs targeting NF-κB activity have been identified and tested for use as a cancer therapeutic [54]. Many of these anti-TNFα therapeutics developed for the treatment of other conditions may more easily studied as a therapeutic agent for PVR, which currently lacks any pharmacologic management options and is associated with poor surgical outcomes [6, 7].

While this work reveals that CSE activated NF-κB signaling to promote TNFα mRNA and protein production in ARPE-19 cells, we did not investigate the mechanism(s) whereby CSE activated NF-κB signaling. NF-κB dependent transcriptional activation is mediated by a family of five related proteins that form a heterodimer of either p50 or p52 and either RELA, c-REL (canonical signaling) or RELB (non-canonical) [55]. Using a knockdown approach, we identified that the RELA subunit of NF-κB is required for TNFα production, suggesting canonical NF-κB activity. Regulation of NF-κB activity is mediated by the degradation of IκB proteins, which usually occurs as a result of extracellular signaling. For example, TNFα signaling itself [24], toll-like receptor (TLR) signaling [56] and epidermal growth factor receptor (EGFR) signaling lead to IκB degradation and NF-κB activation [57]. Recently, Park and Kim showed that CSE induced EGFR activity and FAK signaling to promote EMT in RPE cells [14]. Therefore, it is possible that CSE induces EGFR signaling to promote NF-κB activity. Additionally, once TNFα production is induced, it can form a positive feedback loop leading to additional activation of NF-κB [24, 30]. Finally, TLR signaling has been shown to be disrupted by cigarette smoke in alveolar macrophages [58]. Future investigations studying these pathways in RPE cells will be important to help elucidate the mechanisms driving CSE mediated TNFα production in PVR.

There are a few limitations to our study. Our *in vitro* experiments simulate cigarette smoke exposure, but do not replicate the chronic exposure to cigarette smoke experienced in humans. CSE is widely used as a model system to study *in vitro* effects of tobacco smoke, but this is not without caveats. Although CSE contains many components inhaled by smokers and can readily be taken up by cells [55, 59], this feature makes it difficult to determine the component(s) of cigarette smoke mediating a given biological effect. Additionally, the generation of CSE in aqueous solutions (such as cell culture media) results in the collection of the water-soluble (particulate) components of whole cigarette smoke, which constitute only a fraction of components found in cigarette smoke [60]. However, water-soluble components of cigarette smoke can readily reach systemic circulation [61], suggesting that compounds found in CSE may mimic in vivo situations. Nevertheless, this *in vitro* approach provides insight into the impact of CSE on ARPE-19 cells and has commonly been used in other studies examining the effects of cigarette smoke [35, 55, 60]. Another shortcoming of this work is that our *in vivo* studies utilized injection of CSE cultured RPE cells, which similarly does not replicate the chronicity of cigarette smoke exposure in humans. Comparing PVR development in mice that have been chronically exposed to cigarette smoke is a potential future area of research.

In conclusion, our results demonstrate that a potential aspect of the mechanism of cigarette smoke resulting in a higher risk of PVR development includes activation of EMT in RPE cells through the TNFα/NF-κB/Snail pathway. These findings provide support for further investigation into the levels of TNFα in the vitreous of smokers and non-smokers as well as the role of anti-TNFα pharmacologic agents to treat PVR.

## Supporting information

S1 Data Supporting data file. (XLSX)

S1 File Uncropped western blot images. (PDF)
