# Supplementary material for: TNF-α and NF-κB signaling play a critical role in cigarette smoke-induced epithelial-mesenchymal transition of retinal pigment epithelial cells in proliferative vitreoretinopathy
Source: PLoS One. 2022 Sep 1;17(9):e0271950. doi: 10.1371/journal.pone.0271950 (PMC9436090; doi:10.1371/journal.pone.0271950)
Supplement: S1 File — (PDF) [file pone.0271950.s002.pdf]

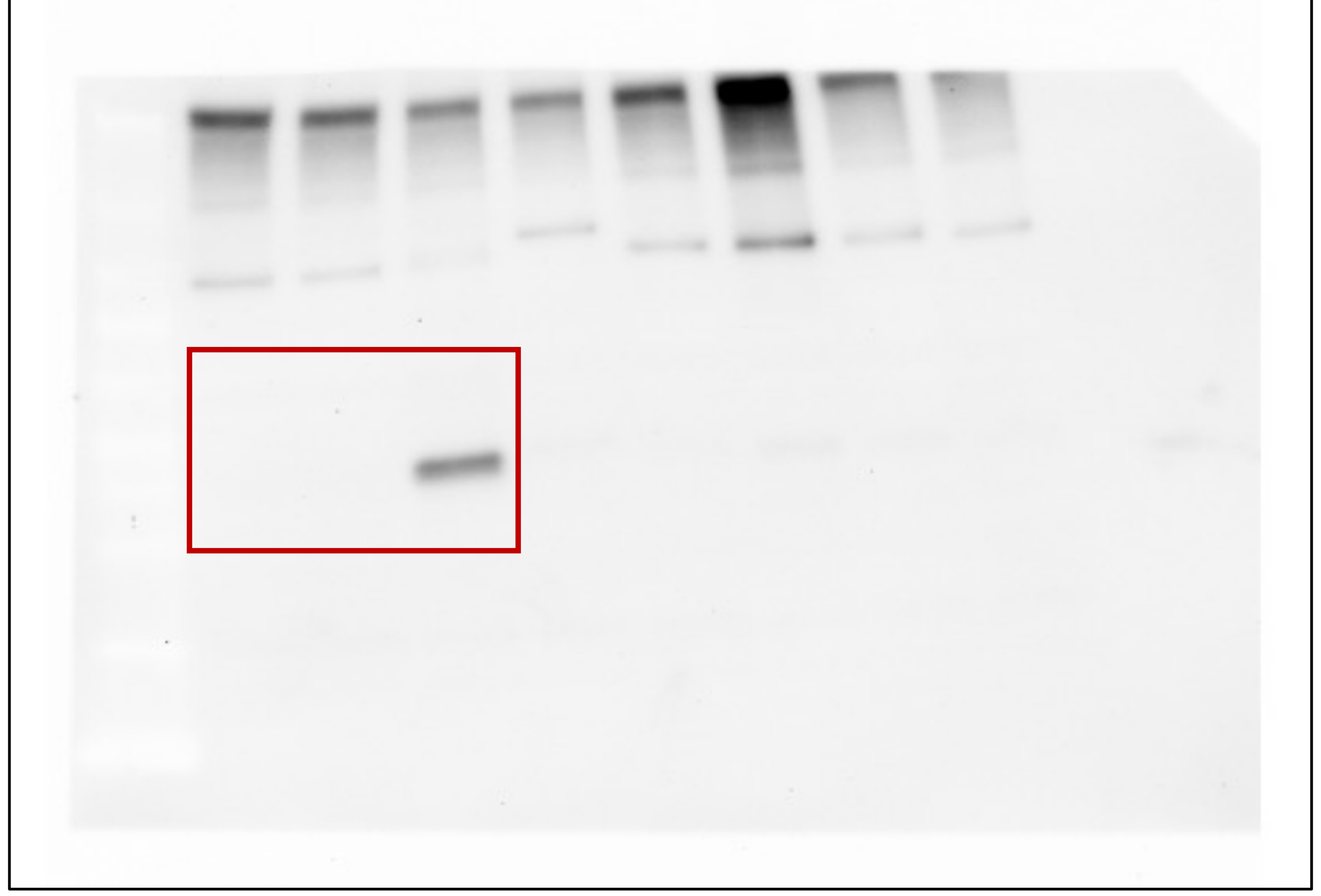

Snail

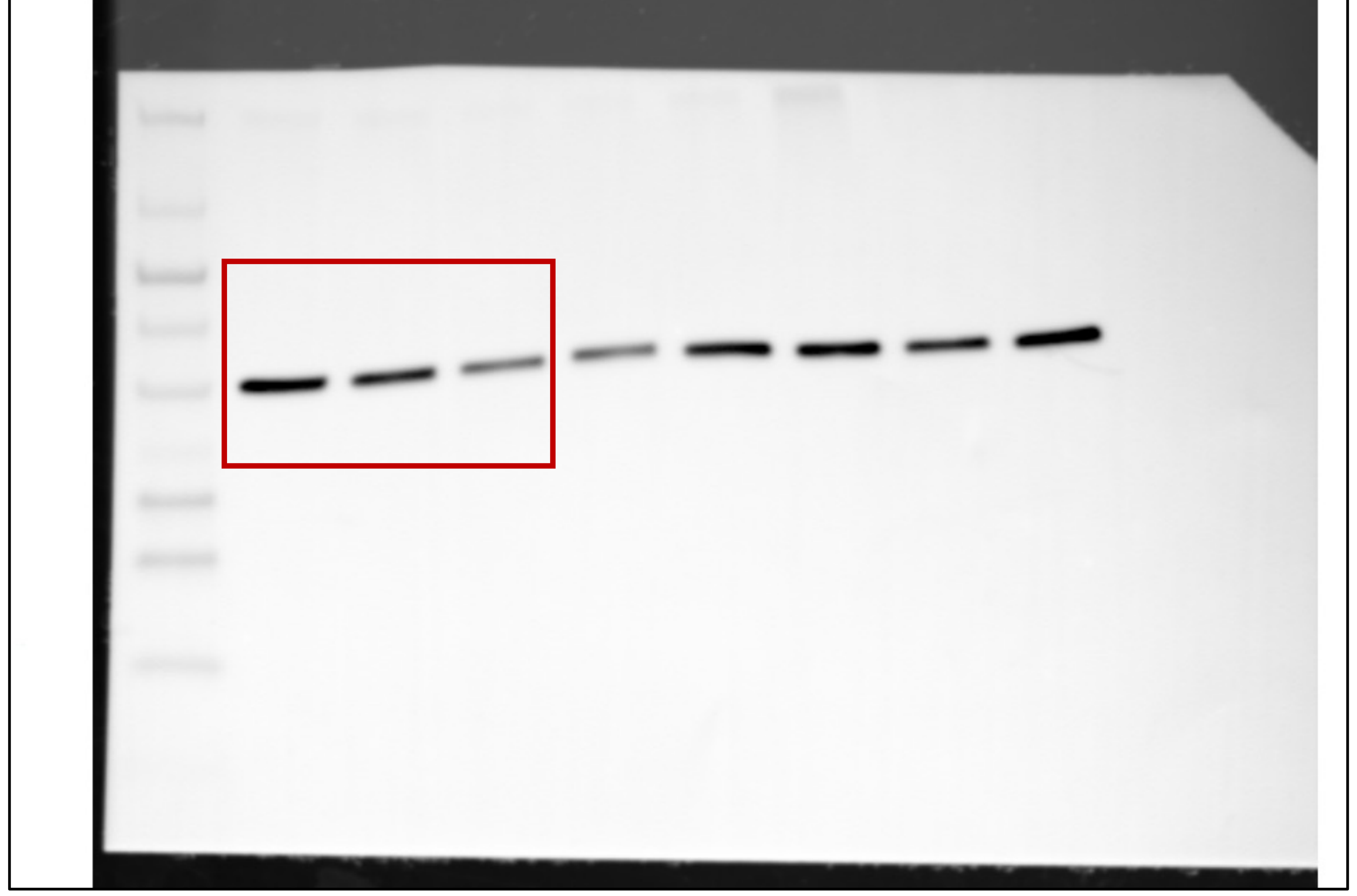

β-tubulin

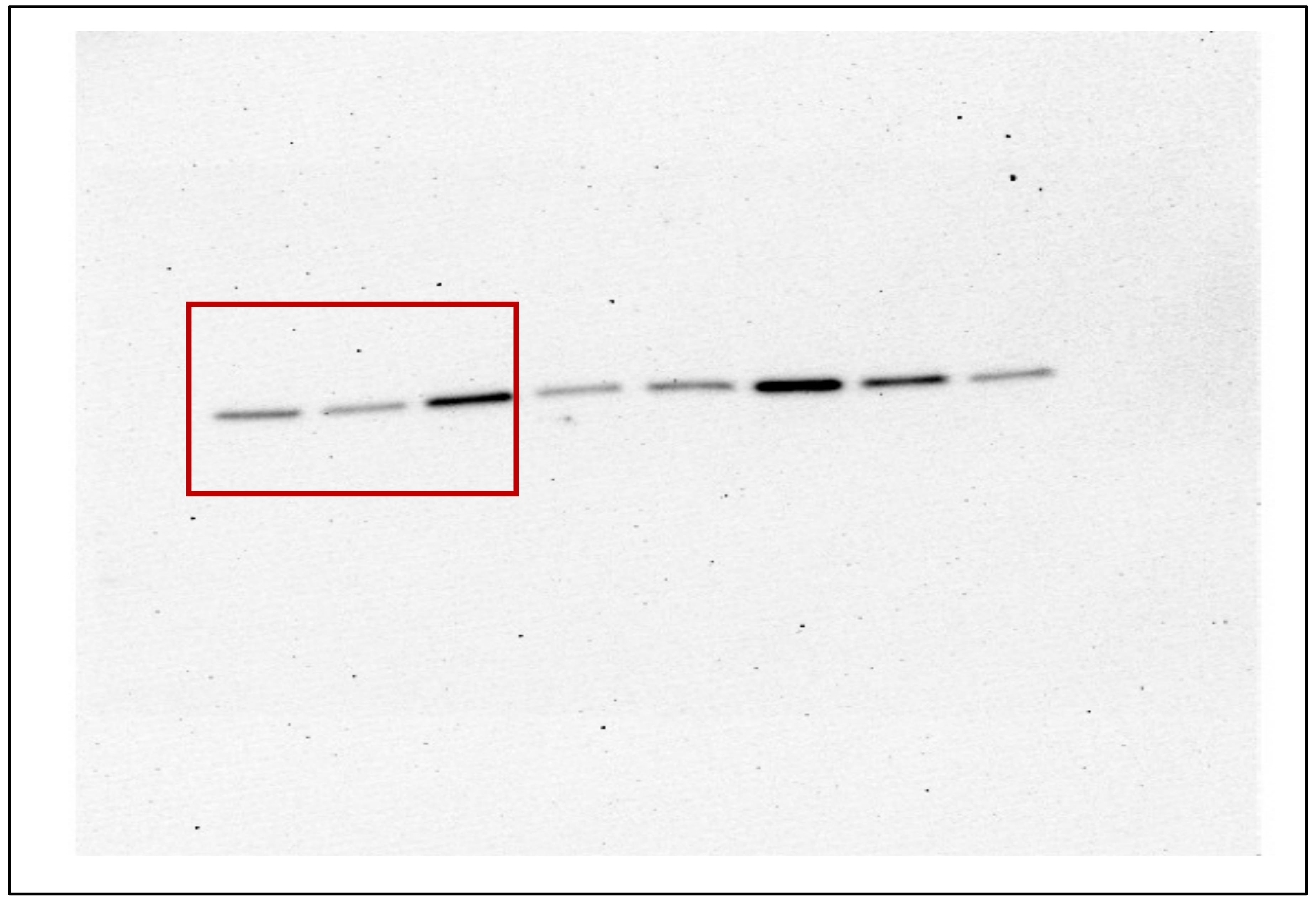

α-SMA

Supplemental Figure 1. Uncropped Western blot images from the blots shown in Figure 1. The red boxes outline the areas presented in Figure 1C.

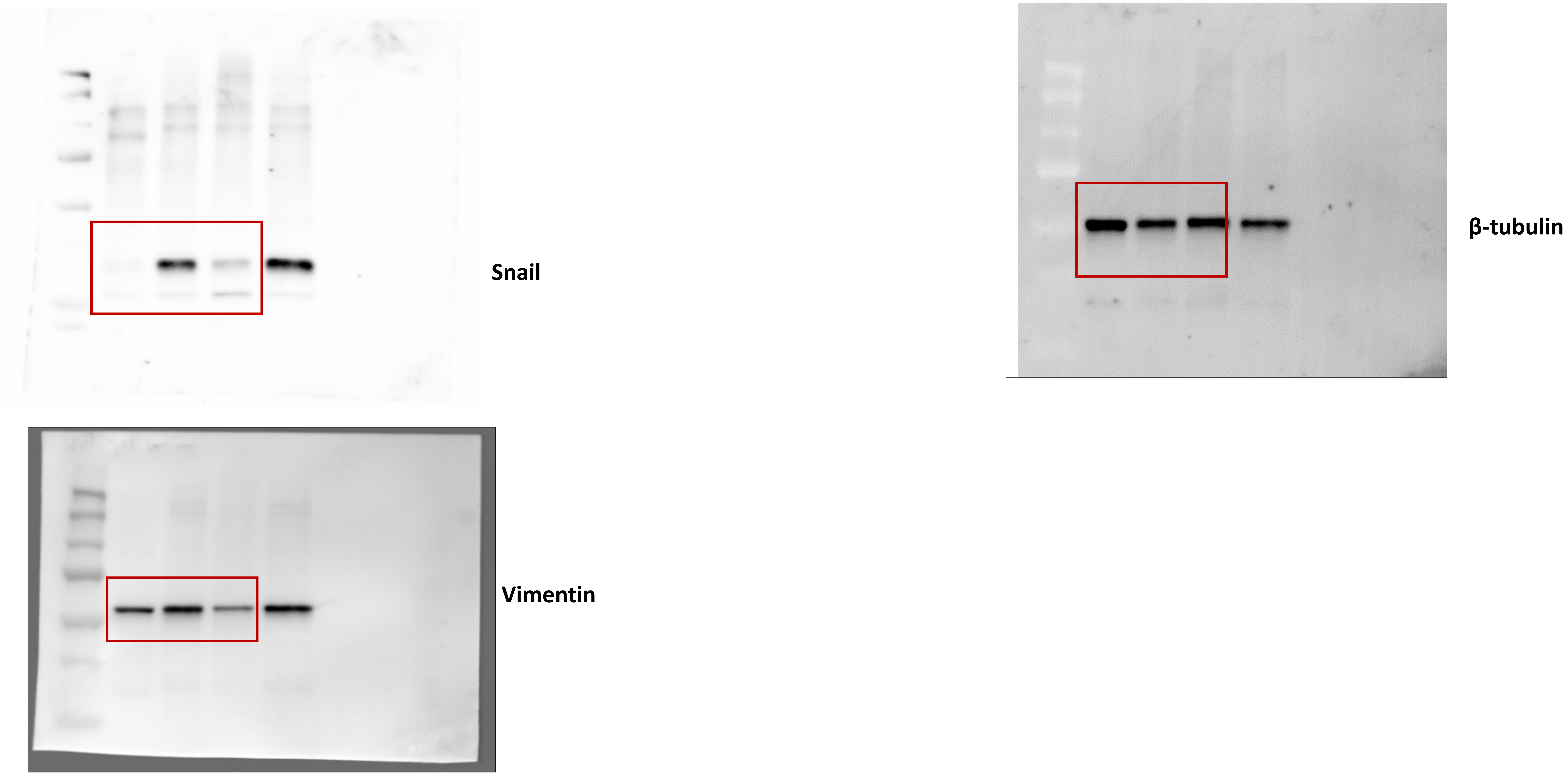

Supplemental Figure 2. Uncropped Western blot images from the blots shown in Figure 1. The red boxes outline the areas presented in Figure 1D.

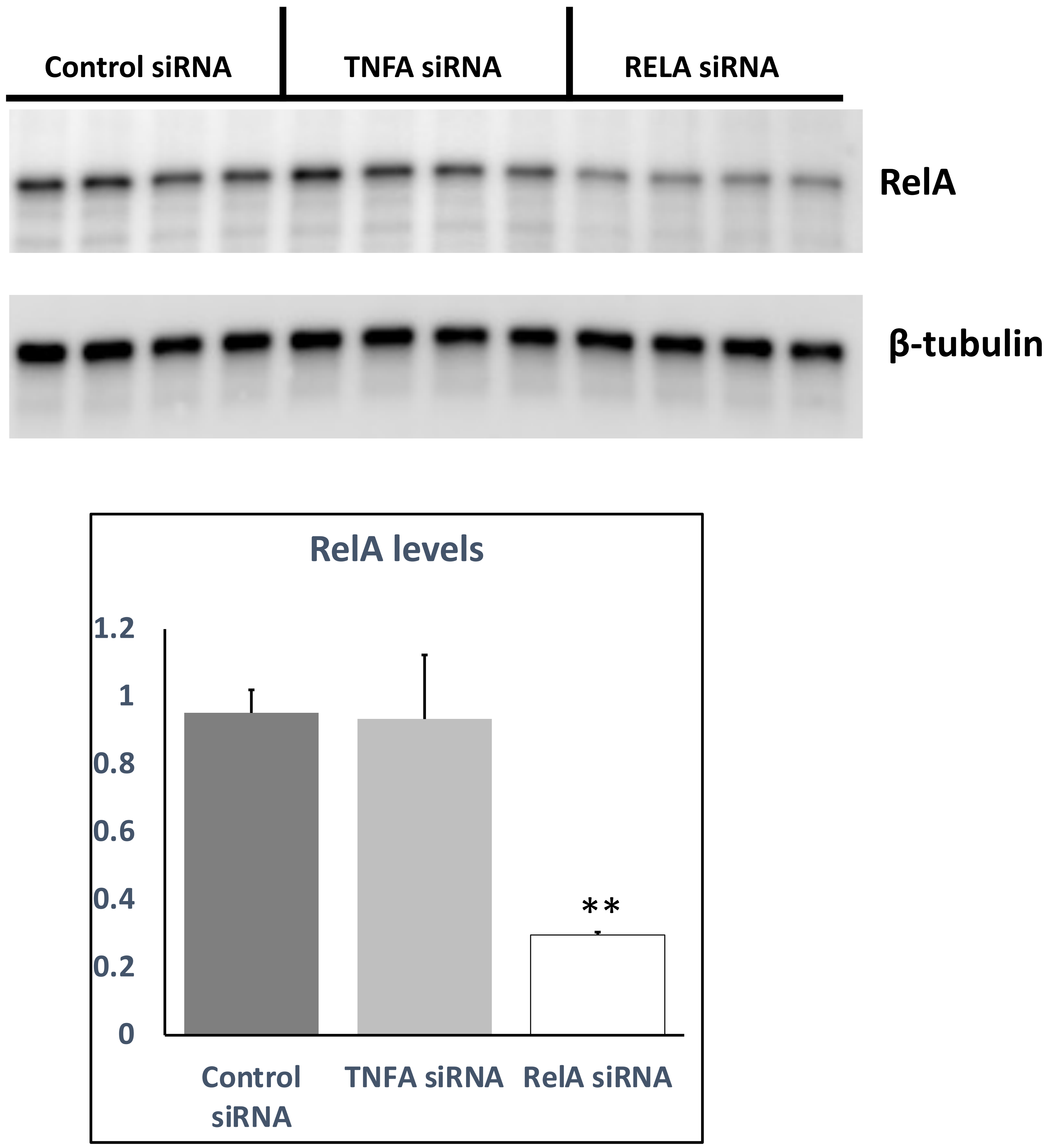

Supplemental Figure 3. Validation of RELA knockdown for experiment in Figure 2F. ** $p < 0.01$.

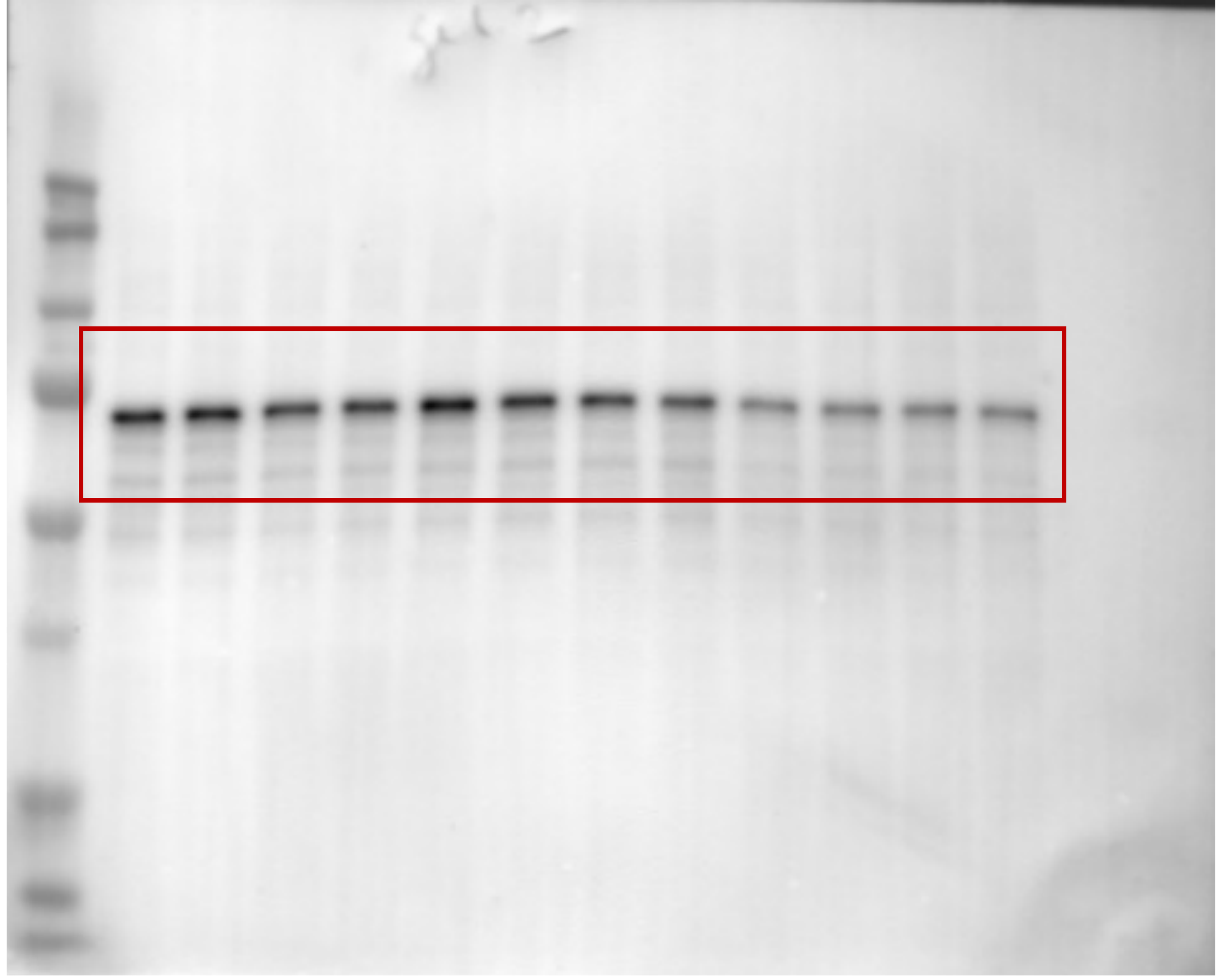

RelA

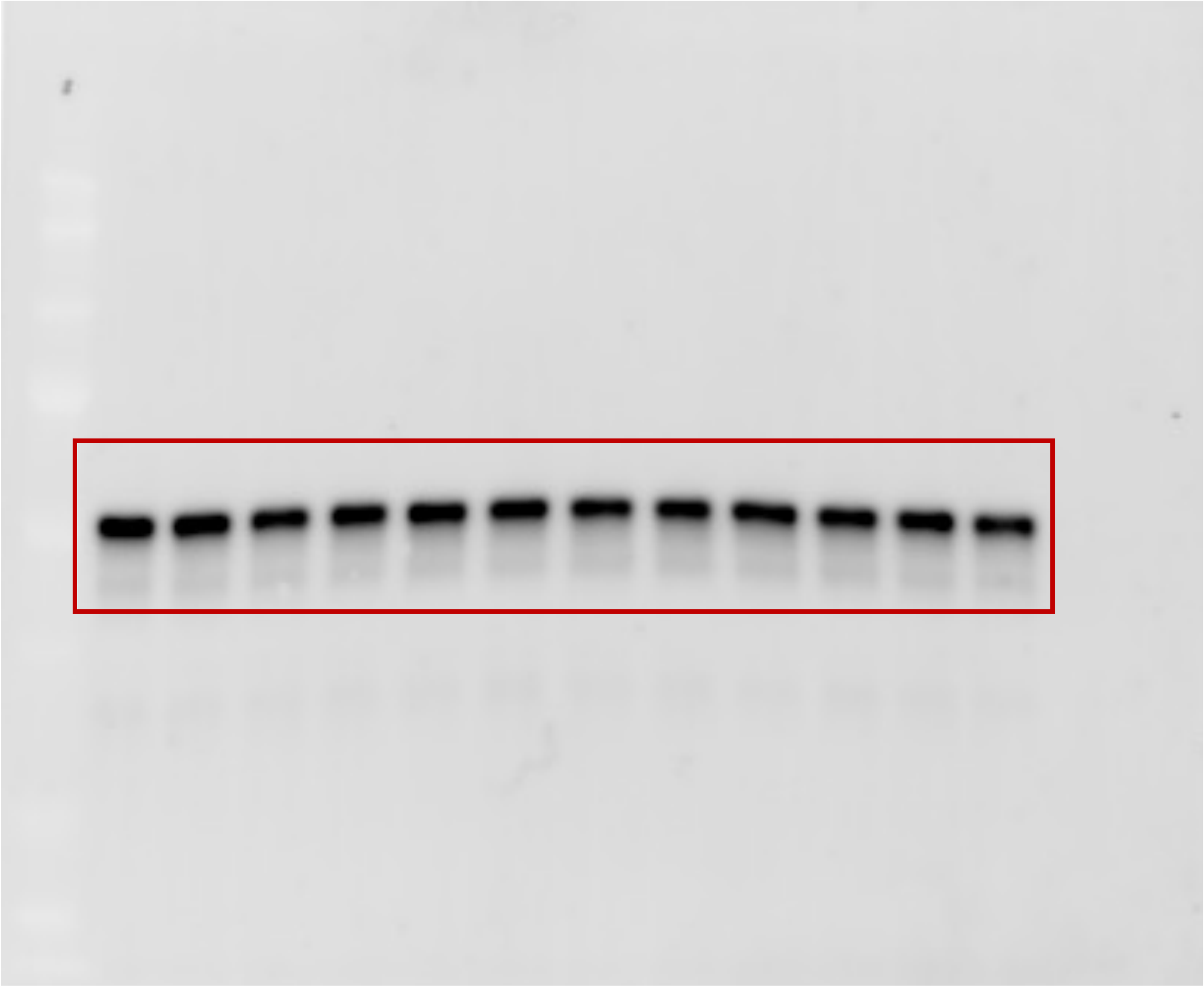

β-tubulin

Supplemental Figure 4. Uncropped Western blot images from the blots shown in Supp Fig 3. The red boxes outline the areas presented in Figure 1D.
